# Discovery of breast cancer risk genes and establishment of a prediction model based on estrogen metabolism regulation

**DOI:** 10.1186/s12885-021-07896-4

**Published:** 2021-02-25

**Authors:** Feng Zhao, Zhixiang Hao, Yanan Zhong, Yinxue Xu, Meng Guo, Bei Zhang, Xiaoxing Yin, Ying Li, Xueyan Zhou

**Affiliations:** 1 Jiangsu Key Laboratory of New Drug Research and Clinical Pharmacy, College of Pharmacy, Xuzhou Medical University, 209 Tongshan Road, Xuzhou, 221004 China; 2 Department of Pharmacy, The First People’s Hospital of Yancheng, The Yancheng Clinical College of Xuzhou Medical University, Yancheng, China; 3 Department of Thyroid and Breast Surgery, The Affiliated Hospital of Xuzhou Medical University, Xuzhou, China; 4 Department of Obstetrics and Gynecology, Xuzhou Central Hospital, Xuzhou Clinical School of Xuzhou Medical University, Xuzhou, China

**Keywords:** Breast cancer, Risk prediction, Estrogens, Estrogen-metabolizing enzyme, Gene polymorphism, Polygenic risk score

## Abstract

**Background:**

Multiple common variants identified by genome-wide association studies have shown limited evidence of the risk of breast cancer in Chinese individuals. In this study, we aimed to uncover the relationship between estrogen levels and the genetic polymorphism of estrogen metabolism-related enzymes in breast cancer (BC) and establish a risk prediction model composed of estrogen-metabolizing enzyme genes and GWAS-identified breast cancer-related genes based on a polygenic risk score.

**Methods:**

Unrelated BC patients and healthy subjects were recruited for analysis of estrogen levels and single nucleotide polymorphisms (SNPs) in genes encoding estrogen metabolism-related enzymes. The polygenic risk score (PRS) was used to explore the combined effect of multiple genes, which was calculated using a Bayesian approach. An independent sample t-test was used to evaluate the differences between PRS scores of BC and healthy subjects. The discriminatory accuracy of the models was compared using the area under the receiver operating characteristic (ROC) curve.

**Results:**

The estrogen homeostasis profile was disturbed in BC patients, with parent estrogens (E1, E2) and carcinogenic catechol estrogens (2/4-OHE1, 2-OHE2, 4-OHE2) significantly accumulating in the serum of BC patients. We then established a PRS model to evaluate the role of SNPs in multiple genes. PRS model 1 (M1) was established from SNPs in 6 GWAS-identified high risk genes. On the basis of M1, we added SNPs from 7 estrogen metabolism enzyme genes to establish PRS model 2 (M2). The independent sample t-test results showed that there was no difference between BC and healthy subjects in M1 (*P* = 0.17); however, there was a significant difference between BC and healthy subjects in M2 (*P* = 4.9*10^− 5^). The ROC curve results showed that the accuracy of M2 (AUC = 62.18%) in breast cancer risk identification was better than that of M1 (AUC = 54.56%).

**Conclusion:**

Estrogen and related metabolic enzyme gene polymorphisms are closely related to BC. The model constructed by adding estrogen metabolic enzyme gene SNPs has a good predictive ability for breast cancer risk, and the accuracy is greatly improved compared with that of the PRS model that only includes GWAS-identified gene SNPs.

## Background

Breast cancer is the most common malignant disease among women worldwide, accounting for 24% of new cancer cases and 15% of cancer deaths in 2018, and incident cases are expected to increase by more than 46% by 2040, according to the GLOBOCAN Cancer Tomorrow prediction tool, which will seriously endanger women’s lives and health [1]. At present, people’s understanding of breast cancer is deepening substantially, and new treatment strategies for tumors, including breast cancer, are continually emerging [2, 3]. With continuous improvements in diagnosis and treatment methods, the survival rate of breast cancer patients has been greatly improved. Early prediction, early detection, and early treatment of high-risk groups are the key issues that urgently need to be solved in the clinic.

The occurrence and development of breast cancer are closely related to genetic and environmental factors. In 1989, Gail proposed the breast cancer risk prediction model, which included factors such as age at evaluation, age at menarche, age at first live birth, race, number of breasts, and family history of breast cancer [4, 5]. Some subsequent prediction models also involved BRCA1/2, estrogen replacement therapy, mammography screening times, and genetic polymorphisms. Rare high-risk mutations, particularly in the BRCA1 and BRCA2 genes, explain less than 20% of the twofold familial relative risk (FRR) and account for a small proportion of breast cancer cases in the general population. Low-frequency variants conferring intermediate risk, such as those in CHEK2, ATM, and PALB2, explain 2 to 5% of the FRR [6]. Genome-wide association studies (GWASs) have led to the discovery of multiple common, low-risk variants (single nucleotide polymorphisms [SNPs]) associated with breast cancer risk [7]. Recently, it was found that genetic risk factors can account for 31% of breast cancer risk evaluations [8], which indicates that breast cancer is a multifactorial disease and that genetic factors are important etiological factors involved in the occurrence and development of breast cancer. At present, an increasing number of researchers are inclined to develop a comprehensive genetic risk scoring method to evaluate the polygenic effects of single nucleotide polymorphisms (SNPs) based on GWASs [9–11]. Some well-known studies, such as Mavaddat et al., used 77 GWAS-selected SNPs to construct a PRS for BC. Compared with middle quintile polygenic scores, the risk scores of the highest 1% were increased threefold [9].

GWASs also have their own limitations. First, a major limitation of genome-wide approaches is the need to adopt a high level of significance to account for multiple tests. Second, GWASs explain only a modest fraction of the missing heritability [12]. Estrogen is an important risk factor for breast cancer. With long-term exposure, super physiological concentrations of estrogen can bind to estrogen receptors, mediate the overexpression of various growth factors, and promote the growth and proliferation of cells, and various metabolites of estrogen can form adducts with DNA, induce genetic mutations and produce direct genotoxicity [13]. Thus, the abnormal accumulation of estrogen and its toxic metabolites in breast tissue is an important risk factor for breast cancer development. Estrogen homeostasis is regulated by estrogen-related metabolic enzymes. Endogenous estrogens are metabolized to be 2-, 4- and 16α-hydroxy estrogens, which are catalyzed by the phase I metabolizing enzymes cytochrome P450 CYP1A1, CYP1B1 and CYP3A4, respectively [14–16]. Hydroxyestrogens are detoxified by conjugation reactions catalyzed by phase II metabolizing enzymes such as COMT, UGTs and SULTs. Thus, the expression level of estrogen and its toxic metabolites can be considered to be a comprehensive reflection of the role of these estrogen metabolic enzymes to a certain extent. Polymorphisms in genes encoding these estrogen-related metabolic enzymes are reported to be closely related to differences in enzyme activities and alter the levels of DNA-damaging species to influence the individual’s susceptibility to breast cancer [14, 17, 18]. Genetic epidemiological studies have suggested that there is a correlation between polymorphisms in estrogen metabolism genes and breast cancer risk; however, these results are not consistent [18–20]. This is an important reason for the inconsistency of existing research results that studied the correlation between gene polymorphisms of estrogen metabolic enzymes and breast cancer in isolation. Currently, breast cancer risk gene prediction models have not taken estrogen metabolic enzyme genes into consideration; therefore, further optimization is needed from the perspective of overall estrogen metabolism levels.

Based on the above analysis, our research aims to reveal the form of estrogen homeostasis disorders in breast cancer and explore the association between metabolic enzyme gene polymorphisms and breast cancer occurrence from the overall level of estrogen metabolism. Furthermore, we developed a risk score comprising GWAS-selected SNPs and estrogen metabolic enzyme gene SNPs to optimize the breast cancer risk prediction model.

## Methods

### Chemicals

The standards and other chemical reagents were described in our previously published study [21].

### Clinical sample collection

Serum samples were collected during the follicular and luteal phases of 64 premenopausal women (mean age: 45.5 ± 5.04 years) first diagnosed with BC and 49 matched healthy women (mean age: 43.7 ± 8.80 years) to detect the level of estrogens. Blood samples were also collected from 140 premenopausal women (mean age: 43.3 ± 6.24 years) first diagnosed with BC and 140 matched healthy women (mean age: 40.2 ± 3.52 years) to extract DNA and analyze SNP genotypes. All samples and related data were obtained from the Affiliated Hospital of Xuzhou Medical University, Xuzhou, China, from June 2017 to May 2019. Patients with BC were enrolled from the Department of Nail Surgery, whereas healthy subjects were enrolled from the physical examination center. Blood samples were collected before any therapy.

The enrollment criteria were as follows: no history of smoking; BMI ranging from 19 to 26; and no history of chemotherapy, radiotherapy, or estrogen-related endocrine therapy during blood sample collection. The characteristics of the patients at baseline can be seen in Table 1. This protocol was approved by the Ethics Committee of the Affiliated Hospital of Xuzhou Medical University. Written informed consent was obtained from each subject before the study.

**Table 1:** The characteristics of the patients at baseline

	Patients	Healthy volunteers
Age (detecting the level of estrogens)	45.5 ± 5.04 years	43.7 ± 8.80
Age (Analyzing SNP genotype)	47.61 ± 3.55 years	40.2 ± 3.52 years
BMI	24.43 ± 3.42	23.09 ± 2.51

*BMI* Body mass index

### Quantification of estrogens using the LC-MS/MS method

The LC-MS/MS method was performed according to our previously published method [21].

### Genotyping analysis

DNA was extracted from peripheral whole blood with a Tiangen DNA extraction kit (Biotech, Beijing, China). The main metabolic enzymes CYP19A1, CYP1A1, CYP1B1, HSD17B1, COMT, UGTs, and SULTs are involved in the regulation of estrogen metabolism. In this study, according to a previous study and pharmacogenomic database, 1 gene locus that is more common or affects the function and activity of metabolic enzymes was screened from each metabolic enzyme. At the same time, we used GWAS-identified breast cancer-related SNPs according to a previous study [22]. All selected SNPs were potentially functional variants, with minor allelic frequencies (MAFs) of more than 10%. The allelic discrimination of the following SNPs was performed by SNaPshot assay (Applied Biosystems Inc., Waltham, MA, USA): estrogen metabolic enzyme gene SNPs including CYP19A1 (rs700519), CYP1A1 (rs1048943), CYP1A1 (rs4646903), CYP1B1 (rs1056827), CYP1B1 (rs1056836), COMT (rs4680), HSD17B1 (rs605059), SULT1A1 (rs1042028), and UGT2B7 (rs7439366) and the GWAS-identified high-risk breast cancer gene SNPs including ZNF365 (rs10822013), FGFR2 (rs2981579), RAD51B (rs3784099), TOX3 (rs3803662), MAP3K1 (rs889312), and HCN1 (rs981782). The allelic discrimination analysis was performed by Genesky Biotechnologies Inc., Shanghai, China (http://www.geneskybiotech.com). Detailed information about the basic SNP information can be found in Table 2. To assure genotyping quality, detailed quality control (QC) procedures, including the duplicate identification of genotypes and a Hardy–Weinberg equilibrium (HWE) test, were carried out. All 15 SNPs were successfully genotyped in 280 subjects with call rates of 100%.

### Statistical analysis

SPSS 22.0 software was used to perform statistical analysis. We used the mean ± SEM to express all estrogen data and Student’s t-test to test differences between the two groups. Multivariate analysis was performed using SIMCA 14.0 software.

HWE was examined among controls using a goodness-of-fit chi-squared test. The odds ratio (OR) and 95% confidence interval (CI) were calculated using a logistic regression model to assess the association between the SNPs and the risk of breast cancer.

We established a PRS to estimate the multigene contribution of estrogen-metabolic enzyme gene loci for breast cancer susceptibility, which was created using marginally significant SNPs associated with breast cancer risk based on the per-allele models. For SNPs in strong linkage disequilibrium located on the same gene or chromosome, we chose the one variant with the lowest *P* value in the per-allele model as a candidate. The basic formula is as follows:

$$ \mathrm{PRS}\kern0.5em =\kern0.5em {\beta}_1{x}_1\kern0.5em +\kern0.5em {\beta}_2{x}_2\kern0.5em +\kern0.5em .\dots \kern0.5em {\beta}_k{x}_k\kern0.5em +\kern0.5em {\beta}_n{x}_n $$

where βk is the per-allele OR for breast cancer associated with the minor allele for SNP k, and xk is the number of alleles for the same SNP (0, 1, or 2).

## Result

### Disorders of estrogen expression in breast cancer patients

**Fig. 1:**
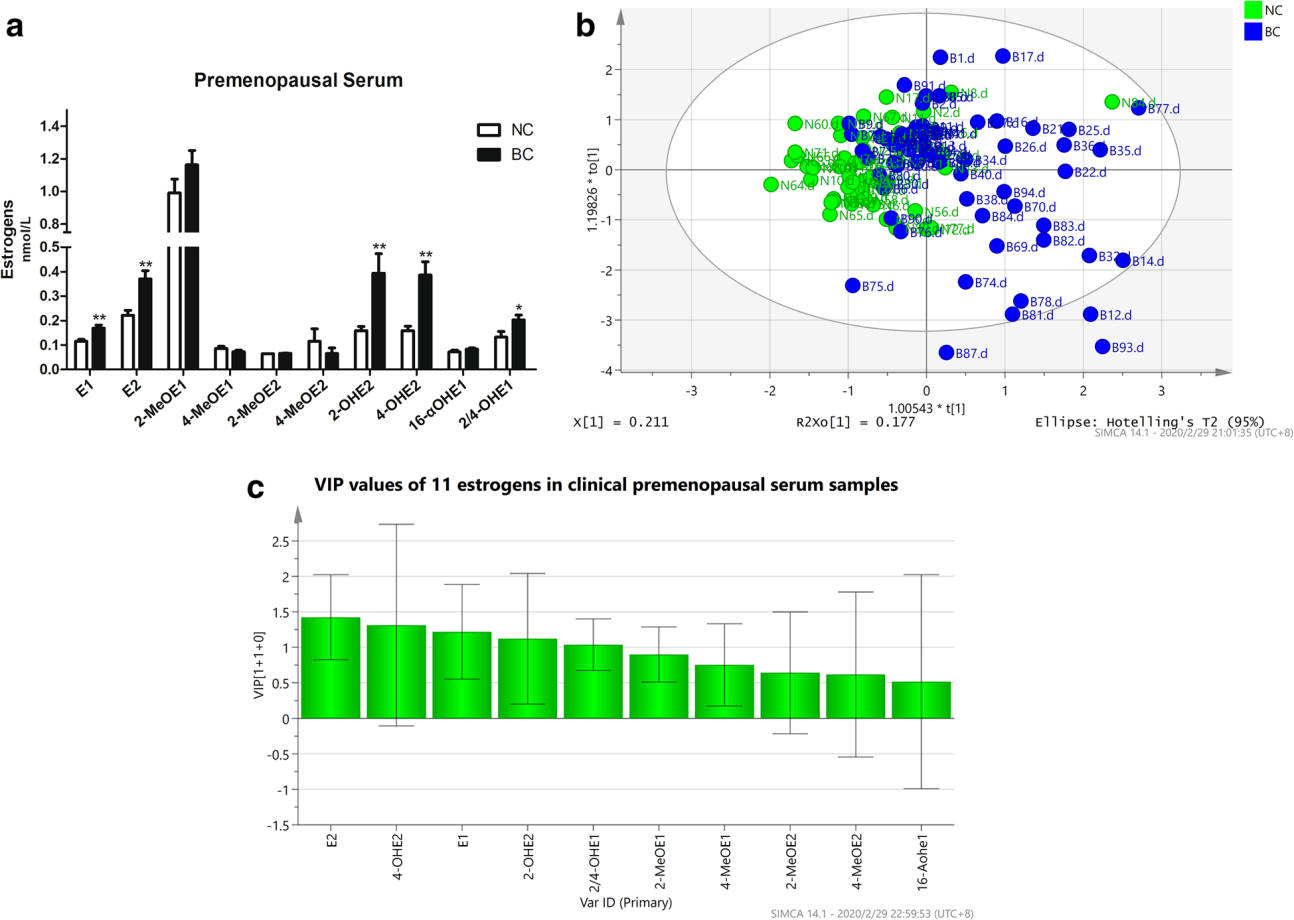
Imbalance of estrogen homeostasis in the serum of BC patients. **a** The concentrations of estrogens, including estrone (E1), estradiol (E2), 16α-hydroxy estrone (16α-OHE1), 2-methoxy estrone (2-MeOE1), 4-methoxy estrone (4-MeOE1), 2-methoxy estradiol (2-MeOE2), 4-methoxy estradiol (4-MeOE2), 2/4-hydroxy estrone (2/4-OHE1), 2-hydroxy estradiol (2-OHE2), and 4-hydroxy estradiol (4-OHE2), in serum samples from NC (49 healthy women, mean age of 43.7 ± 8.80 years) and BC (64 breast cancer patients, mean age of 45.5 ± 5.04 years) were detected by LC-MS/MS. *, *p* < 0.05, **, *p* < 0.01 vs control group. The results are shown as mean ± SEM values to depict the levels of estrogens in serum of BC patients and healthy women. **b** Orthogonal Projections to Latent Structures-Discriminant Analysis (OPLS-DA) score plots of serum (R2X(cum) = 0.335, R2Y(cum) = 0.264, Q2(cum) = 0.003) estrogen metabolites in NC group (green) and BC group (blue) generated by SIMCA 14.0 software. **c** Variable importance in the projection (VIP) values calculated from OPLS-DA models for estrogen metabolic profile data

Using LC-MS/MS quantitative analysis, we measured the expression levels of 11 serum estrogens and metabolites in 64 patients with premenopausal BC (mean age: 45.5 ± 5.04 years) and 49 matched controls (mean age: 43.7 ± 8.80 years). We found that there was no significant difference in age between the BC group and NC group. As shown in Fig. 1a, compared with the NC group, the BC group exhibited significantly increased estrogen levels, especially E1, E2, 2-OHE2, 4-OHE2 (*P* < 0.01) and 2/4-OHE1 (*P* < 0.05). OPLS-DA was constructed as an unsupervised statistical method to identify potential estrogen homeostatic changes between the two groups. As shown in Fig. 1b, the metabolic profile of the NC group was clearly separated from that of the BC group, indicating that there was a considerable metabolite difference between the BC group and NC group. We also found that the potential biomarkers with VIP values higher than 1.0 in the OPLS-DA model were E1, E2, 2-OHE2, 4-OHE2 and 2/4-OHE1 in the serum of BC patients (Fig. 1c). Overall, these results supported the view that the disorder of estrogen homeostasis was closely related to increased risk of BC.

### Cohort description and Hardy–Weinberg equilibrium testing

We enrolled 140 patients first diagnosed with breast cancer and 140 corresponding healthy women in this study. The mean age at diagnosis (for patients with cancer) was 43.3 ± 6.24 years, and the mean age of healthy women at enrollment was 40.2 ± 3.52 years. Blood samples were collected from these participants to extract DNA and analyze the SNP genotype. We found that there was no significant difference in age between the BC group and NC group. The chi-square test was used to test the HWE value, and *P* > 0.05 explained that the samples at enrollment were representative of the group. As seen in Table 2, all polymorphisms were found to be in genetic equilibrium, which indicated that the observed genotype frequencies of the case and control groups were constant and representative.

**Table 2:** The basic information and HWE testing of each estrogens metabolizing enzymes gene polymorphisms

Gene	rs number	Chromosome position	Domain	Alleles	Amino acid change	Metabolism estrogens	Test for HWE (***p***)
CYP19A1	rs700519	Chr15: 51507968	exon7	G/A	Arg264Cys	E1(E2)	0.392
CYP1A1	rs1048943	Chr15: 75012985	exon7	T/C	Ile462Val	2-OHE1(E2)	0.241
CYP1A1	rs4646903	Chr15: 75011641	3′-flanking	A/G	/	2-OHE1(E2)	1.000
CYP1B1	rs1056827	Chr2: 38302177	exon2	C/A	Ala119Ser	4-OHE1(E2)	0.602
CYP1B1	rs1056836	Chr2: 38298203	exon3	G/C	Val432Leu	4-OHE1(E2)	0.101
HSD17B1	rs605059	Chr17: 40706906	exon6	G/A	Gly313Ser	E2	0.106
COMT	rs4680	Chr22: 19951271	exon4	G/A	Val158Met	2 (4)-MeOE1(2)	1.000
SULT1A1	rs1042028	Chr16: 28617514	exon7	C/T	Arg213His	Sulfated metabolites	0.144
UGT2B7	rs7439366	Chr4: 69964338	exon2	C/T	Tyr268His	Glucuronide metabolites	0.086
ZNF365	rs10822013	Chr10: 64251977	intron4	C/T	/	/	0.478
FGFR2	rs2981579	Chr10: 123337335	intron2	G/A	/	/	0.665
CASC16	rs3803662	Chr16: 52586341	exon4	G/A	/	/	0.360
RAD51B	rs3784099	Chr14: 68749927	intron7	G/A	/	/	0.456
MAP3K1	rs889312	Chr5: 56031884	/	A/C	/	/	0.776
HCN1	rs981782	Chr5: 45285616	intron6	A/C	/	/	0.818

*HWE* Hardy–Weinberg equilibrium

### Association of estrogen-metabolizing enzyme genetic variants with breast cancer risk

Table 3 shows univariate analysis and ORs related to each metabolizing enzyme SNP. The polymorphic genotypes of CYP1A1 rs1048943 (*P* = 0.007), CYP1B1 rs1056827 (*P* = 0.004), CYP1B1 rs1056836 (*P* = 0.002) and SULT1A1 rs1042028 (*P* = 0.029) showed significant differences in distribution. Compared with the wild-type genotypes of CYP1A1 rs1048943 (TT) or SULT1A1 rs1042028 (CC), the heterozygous variant genotypes of CYP1A1 rs1048943 (TC) or SULT1A1 rs1042028 (CT) showed significantly higher risk in breast cancer, with ORs of 2.37 (95% confidence interval [CI] = 1.27–4.43) and 2.21 (95% CI = 1.20–4.05), respectively. Compared with the wild-type genotypes of CYP1B1 rs1056827 (CC), the homozygous variant genotypes (AA) showed a significantly higher risk in breast cancer, yielding an OR of 6.90 (95% CI = 1.50–31.76). Compared with the wild-type genotypes of CYP1B1 rs1056836 (GG), the heterozygous variant genotypes significantly reduced the risk of breast cancer, yielding an OR of 0.37 (95% CI = 0.21–0.67). In addition, no associations with breast cancer risk were observed for the estrogen metabolic enzyme gene SNPs CYP19A1 (rs700519), HSD17B1 (rs605059), COMT (rs4680), or UGT2B7 (rs7439366) or the GWAS-selected SNPs ZNF365 (rs10822013), FGFR2 (rs2981579), RAD51B (rs3784099), TOX3 (rs3803662), MAP3K1 (rs889312), or HCN1 (rs981782).

**Table 3:** Genotype frequencies and ORs associated with each gene polymorphism in breast cancer cases and controls

Gene and SNPs	Genotype	Control n (%)	Case n (%)	***P***-value^**#**^	OR (95% CI)	***P*** –value*
CYP19A1 (rs700519)	GG	97 (69.3%)	92 (65.7%)	0.813	1	–
	GA	37 (26.4%)	41 (29.3%)		1.17 (0.69–1.98)	0.564
	AA	6 (4.3%)	7 (5.0%)		1.23 (0.40–3.80)	0.719
CYP1A1 (rs1048943)	TT	100 (71.4%)	80 (57.1%)	0.007	1	–
	TC	31 (22.2%)	55 (39.3%)		2.37 (1.27–4.43)	0.003
	CC	9 (6.4%)	5 (3.6%)		1.10 (0.30–4.00)	0.528
CYP1A1 (rs4649903)	AA	68 (48.6%)	58 (41.4%)	0.300	1	–
	AG	56 (40.0%)	58 (41.4%)		1.21 (0.73–2.02)	0.453
	GG	16 (11.4%)	24 (17.1%)		1.76 (0.85–3.62)	0.126
CYP1B1 (rs1056827)	CC	92 (65.7%)	80 (57.1%)	0.004	1	–
	CA	48 (34.3%)	50 35.7%)		1.20 (0.73–1.97)	0.802
	AA	0 (0.0%)	10 (7.2%)		6.90 (1.50–31.76)	0.001
CYP1B1 (rs1056836)	GG	90 (64.3%)	116 (82.9%)	0.002	1	–
	GC	44 (31.4%)	21 (15.0%)		0.37 (0.21–0.67)	0.001
	CC	6 (4.3%)	3 (2.1%)		0.39 (0.10–1.59)	0.189
HSD17B1 (rs605059)	GG	47 (33.6%)	46 (32.9%)	0.713	1	–
	GA	73 (52.1%)	69 (49.3%)		0.97 (0.57–1.63)	0.896
	AA	20 (14.3%)	25 (17.8%)		1.28 (0.63–2.61)	0.502
COMT (rs4680)	GG	91 (65.0%)	80 (57.1%)	0.402	1	–
	GA	42 (30.0%)	51 (36.4%)		1.38 (0.83–2.29)	0.212
	AA	7 (5.0%)	9 (6.4%)		1.46 (0.52–4.11)	0.470
SULT1A1 (rs1042028)	CC	117 (83.6%)	98 (70.0%)	0.029	1	–
	CT	20 (14.3%)	37 (26.4%)		2.21 (1.20–4.05)	0.010
	TT	3 (2.1%)	5 (3.6%)		1.99 (0.46–8.54)	0.354
UGT2B7 (rs7439366)	CC	69 (49.30%)	64 (45.7%)	0.824	1	–
	CT	60 (42.80%)	65 (46.4%)		1.17 (0.72–1.90)	0.533
	TT	11 (7.90%)	11 (7.90%)		1.08 (0.44–2.66)	0.870
ZNF365 (rs10822013)	CC	36 (25.71%)	43 (30.71%)	0.640	1	–
	CT	75 (53.57%)	71 (50.71%)		0.79 (0.46–1.37)	0.407
	TT	29 (20.71%)	26 (18.57%)		0.75 (0.38–1.50)	0.415
FGFR2 (rs2981579)	GG	47 (33.57%)	40 (28.57%)	0.418	1	–
	GA	70 (50.00%)	69 (49.29%)		1.16 (0.68–1.98)	0.592
	AA	23 (16.43%)	31 (22.14%)		1.58 (0.80–3.14)	0.188
RAD51B (rs3784099)	GG	111 (79.29%)	109 (77.86%)	0.848	1	–
	GA	25 (17.86%)	28 (20.00%)		1.14 (0.63–2.08)	0.668
	AA	4 (2.86%)	3 (2.14%)		0.76 (0.17–3.49)	0.728
TOX3 (rs3803662)	GG	15 (10.71%)	18 (12.86%)	0.664	1	–
	GA	61 (43.57%)	54 (38.57%)		0.83 (0.51–1.38)	0.475
	AA	64 (45.71%)	68 (48.57%)		1.13 (0.53–2.43)	0.755
MAP3K1 (rs889312)	CC	42 (30.00%)	35 (25.00%)	0.460	1	–
	CA	67 (47.86%)	66 (47.14%)		1.18 (0.67–2.08)	0.560
	AA	31 (22.14%)	39 (27.86%)		1.51 (0.79–2.89)	0.215
HCN1 (rs981782)	CC	16 (11.43%)	25 (17.86%)	0.475	1	–
	CA	69 (49.29%)	63 (45.00%)		0.92 (0.55–1.53)	0.737
	AA	55 (39.29%)	52 (37.14%)		1.45 (0.68–3.08)	0.336

Values are presented as number (%) or OR (95% CI)

*OR* Odds radio, *CI* Confidence interval, *SNP* Single nuclear polymorphism

#Comparison of polymorphic genotype distributions in patients with breast cancer and healthy case-controls

*Comparison of wild-type genotypes with heterozygous genotypes and homozygous variant genotypes respectively

### PRS breast cancer risk prediction model establishment and evaluation

**Fig. 2:**
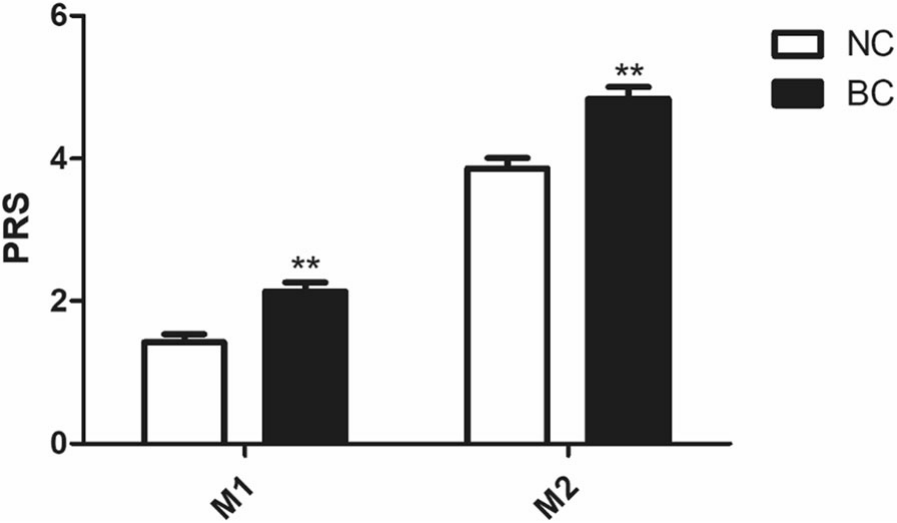
The Polygenic Risk Scores (PRS) of the NC group and BC group in the two risk gene models: PRS model 1 (M1) and PRS model 2 (M2). The results are shown as mean ± SEM values to depict the distribution difference between NC and BC. *, *p* < 0.05, **, *p* < 0.01 vs control group

**Fig. 3:**
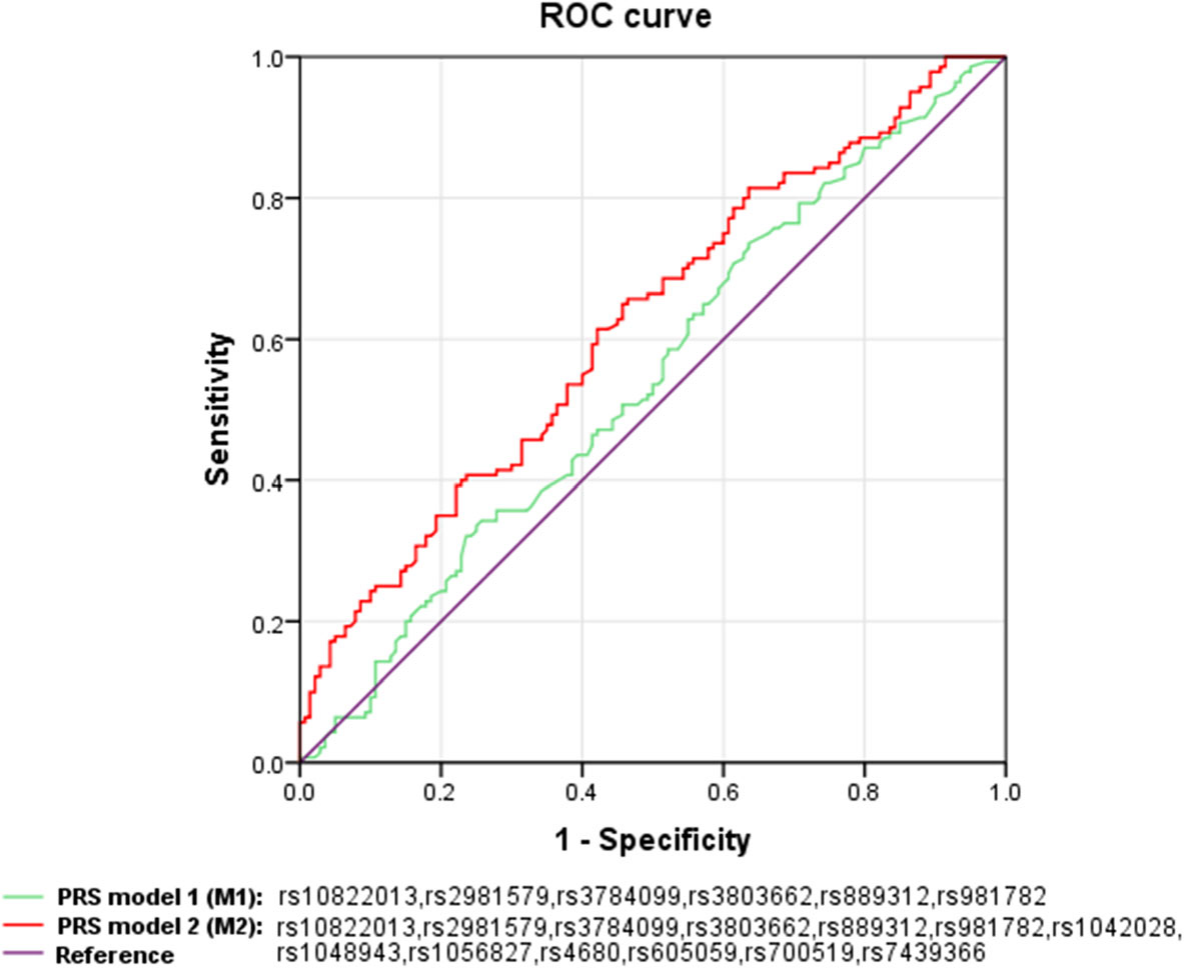
The receiver operating characteristic (ROC) curve in the two risk models. The purple line with an area under the ROC curve (AUC) of 50% is the reference. The AUC of the upper red line, which showed the PRS model 2 (M2), is 62.18%. The green line with a ROC of 54.56% is PRS model 1 (M1)

The binary logistic regression method was used to calculate the OR of the per-allele model, and the detailed results are shown in Table 4. We used SNPs in the GWAS-identified high breast risk genes, namely, ZNF365 (rs10822013), FGFR2 (rs2981579), RAD51B (rs3784099), TOX3 (rs3803662), MAP3K1 (rs889312), and HCN1 (rs981782), to create PRS model 1 (M1) in the per-allele model. On the basis of M1, we also added estrogen metabolic enzyme gene SNPs, namely, CYP1A1 (rs1048943), CYP1B1 (rs1056827), SULT1A1 (rs1042028), CYP19A1 (rs700519), COMT (rs4680), HSD17B1 (rs605059), and UGT2B7 (rs7439366), to create PRS model 2 (M2). For SNPs in strong linkage disequilibrium located on the same gene or chromosome, we chose the one variant (rs1048943) with the lowest *P* value in CYP1A1, and rs1056836 is a protective gene loci, we chose the risk variant rs10526827 in CYP1B1. The PRS scores are expressed as the means ± SEM to find the difference between the two groups. Under M1 and M2, the PRS data of the two groups obeyed a normal distribution; therefore, we used an independent sample t-test to evaluate the difference between the two groups of data. As shown in Table 5 and Fig. 2, the PRS scores in the NC group were significantly lower than those in the BC group in M2 (*P* = 4.9*10^− 5^); however, there was no significant difference between NC and BC in M1 (*P* = 0.17). Finally, the ROC curve was calculated to evaluate how the risk models discriminated between women with and without breast cancer (Fig. 3). The ROC curve estimated for M2 was 62.18% (95% confidence interval [CI] = 0.56–0.69), whereas that for M1 was only 54.56% (95% confidence interval [CI] = 0.48–0.61). Therefore, the accuracy of M2 in breast cancer risk identification was better than that of M1.

**Table 4:** Univariate analysis and ORs associated with Per-allele model

Gene name	SNP rs number	Allele Risk/reference	OR^**a**^ (95% CI)	***P*** value^*^
			Per-allele	
CYP19A1	rs700519	G/A	1.15 (0.75–1.77)	0.515
CYP1A1	rs1048943	C/T	1.43 (0.94–2.16)	0.094
CYP1B1	rs1056827	A/C	1.61 (1.07–2.43)	0.023^*^
HSD17B1	rs605059	G/A	1.09 (0.78–1.53)	0.607
COMT	rs4680	G/A	1.31 (0.88–1.90)	0.188
SULT1A1	rs1042028	T/C	1.97 (1.18–3.29)	0.009^*^
UGT2B7	rs7439366	T/C	1.09 (0.76–1.56)	0.645
ZNF365	rs10822013	C/T	0.87 (0.62–1.21)	0.396
FGFR2	rs2981579	G/A	1.24 (0.89–1.74)	0.202
RAD51B	rs3784099	G/A	1.03 (0.62–1.72)	0.896
TOX3	rs3803662	G/A	0.98 (0.69–1.40)	0.928
MAP3K1	rs889312	C/A	1.00 (0.72–1.39)	1.000
HCN1	rs981782	G/A	1.20 (0.85–1.69)	0.297

*Comparison in Per-allele model

**Table 5:** PRS value results and difference analysis of two gene combinations (M1 and M2)

Model	Group	PRS (Mean ± SEM)	Data distribution	Testing method	***P*** value
M1	NC group	4.52 **±** 0.15	Normal distribution	T-test	**0.17**
	BC group	4.80 **±** 0.14			
M2	NC group	8.38 **±** 0.21	Normal distribution	T-test	4.90*10^**−5**^
	BC group	9.63 **±** 0.22

## Discussion

Breast cancer (BC) is an estrogen-dependent tumor, and the occurrence of BC is closely related to the imbalance of estrogen homeostasis. The accumulation of estrogen and its toxic metabolites in vivo is a significant risk factor for BC development. Different types of estrogens have different physiological and pathological activities and can play an important role in the process of cancer development through different mechanisms. Parent estrogens are postulated to promote tumorigenesis directly through the stimulation of the estrogen receptor (ER) [23]. The endogenous conversion of estrogen to genotoxic metabolites has been reported as an alternative, potentially ER-independent mechanism for estrogen-dependent breast tumorigenesis [24]. Catechol estrogens can form adducts with DNA, causing gene mutations and producing direct genotoxicity [13]. Methoxyestrogens, including 2-methoxyestradiol, have been shown to inhibit carcinogenesis by suppressing cell proliferation and estrogen oxidation due to their effects on microtubule stabilization [25].

In this study, the LC-MS/MS quantitative analysis method was used to determine the serum estrogens in the BC group and NC group. Comparing the levels of serum estrogens in the follicular phase and luteal phase of premenopausal breast cancer patients with healthy female volunteers, we found that the levels of parent and hydroxylated estrogen in the BC group were significantly higher than those in the NC group, which indicated that estrogen metabolism disorder is closely related to the occurrence and development of breast cancer. Using OPLS-DA, we also noticed that E1, E2, 4-OHE2, 2-OHE2, and 2/4-OHE1 are BC-related disease markers. This result was consistent with the epidemiologic characteristics of patients with BC [26].

A large number of studies have confirmed that breast cancer exhibits heritability [27, 28]. However, high-risk genes such as BRCA1 and BRCA2 account for less than 15% of breast cancer cases [29, 30], which suggests that numerous breast cancer-related risk genes have not been discovered, and these gene polymorphisms influence susceptibility to breast cancer.

Estrogen is an important risk factor for breast cancer. However, no research has incorporated estrogens into the breast cancer risk prediction model. A possible major reason is that there is no clinically effective estrogen evaluation method because the steady state of estrogen is affected by various physiological and pathological factors, such as menstrual cycle fluctuations. However, estrogen homeostasis is regulated by various metabolic enzymes. Therefore, we believe that estrogen metabolic enzyme gene polymorphisms are closely related to estrogen homeostasis and the occurrence and development of breast cancer. In this study, univariate logistic regression analysis showed that CYP1A1, CYP1B1, and SULT1A1 gene polymorphisms are closely related to the occurrence of breast cancer. It is worth noting that these gene polymorphisms are also associated with other estrogen-dependent tumors such as endometrial cancer and ovarian cancer. Hiroshi Hirata et al. found that the SULT1A1 rs9282861 (rs1042028) was related to endometrial cancer [31]. A meta-analysis was performed to research the association between CYP1A1 gene polymorphism and ovarian cancer risk, which showed that the Ile/Val (rs1048943) was significantly associated with ovarian cancer, with homozygous carriers (Val/Val vs. Ile/Ile: OR = 2.64; 95% CI: 1.63–4.28) being risk factors for ovarian cancer development [32].

CYP1A1 and CYP1B1 are the major phase I drug metabolism enzymes that catalyze the hydroxylation of estrogens. The increasing polarity of estrogens may be related to the risk of breast cancer [14]. Our experiments also verified this view. In this study, we found that the variant allele of CYP1B1 rs1086836 was involved in reducing the risk of breast cancer and that the exact mechanism of the protection of this variant allele was not clear [33]. We assumed that the heterozygous model of CYP1B1 rs1086836 (GC vs. GG: OR = 0.37, 95% CI: 0.21–0.67, *P* = 0.001) may result in decreased function of the CYP1B1 enzyme, reducing the production of 4-hydroxy estrogen and even catechol estrogen-3,4-quinone (CE-3,4-Q) to form adducts with DNA. At the same time, this study also proved that the variant alleles of CYP1A1 rs1048943 (TC vs. TT: OR = 2.37, 95% CI: 1.27–4.43, *P* = 0.003) and CYP1B1 rs1056827 (AA vs. CC: OR = 6.90, 95% CI: 1.50–31.76, *P* = 0.001) are closely related to the risk of breast cancer, which is consistent with most research [34, 35]. The possible reason is that the mutations promote the activity of CYP1A1 and CYP1B1 enzymes to increase the production of hydroxylated estrogens or promote the individual’s susceptibility to estrogen.

SULTs catalyze the sulfate conjugation of a broad range of substrates and play an important role in the metabolism of endogenous and exogenous compounds, including thyroid and steroid hormones, neurotransmitters, drugs and procarcinogens [36]. SULTs catalyze the sulfated metabolism of estrogen (E1 and E2) and its metabolites (such as catechol estrogen) and eliminate the activity of estrogen by forming sulfate compounds: sulfated estrogens that cannot combine with estrogen receptors (ERs). At the same time, it promotes the rapid excretion of sulfated estrogen from the cells, which can reduce the level of estrogen exposure in the circulation and target tissues. SULT1A1 rs1042028 is the most widely studied gene polymorphism. Its allelic variation can reduce enzyme activity and thermal stability, resulting in increased estrogen accumulation and increased individual susceptibility to breast cancer [37]. In this study, the heterozygous model of rs1042028 had a 2.21-fold higher risk of breast cancer than the wild-type model. This is consistent with the results of multiple studies [38, 39].

Previous studies investigated associations between the PRS of multiple SNPs and breast cancer risk to study the cumulative effect of genes on the disease. Mavaddat et al. constructed a 77-SNP PRS for breast cancer and found a threefold increase in risk when comparing the polygenic scores of the highest 1% and the middle quintiles [9]. Harlid et al. investigated the combined effect of low-penetrant SNPs on breast cancer, which included ten SNPs, and found that the cumulative effect was strongly correlated with breast cancer [40]. However, most of this research on PRS comes from the Caucasian population sample database. Although Sueta, Chan and others have also conducted similar studies in Asian populations, the evidence is still limited [7, 41]. To date, there have been no relevant reports on the establishment of a breast cancer PRS risk prediction model from the perspective of estrogen-metabolizing enzymes. Thus, a multigene PRS model including estrogen metabolic enzyme gene SNPs and GWAS-selected SNPs was constructed in this study to evaluate the comprehensive effects of multiple estrogen metabolic enzyme SNPs on breast cancer.

In this study, we evaluated possible relationships between the increased breast cancer risk estrogen metabolic enzyme gene SNPs and GWAS-identified gene SNPs in an Asian population. Among them, the GWAS-identified SNPs were not associated with breast cancer risk in the per-allele model or dominant model in our study. This finding was inconsistent with a previous study [23]. Further, we established PRS model 1, including only GWAS-identified SNPs, and PRS model 2, which included estrogen metabolic enzyme gene SNPs on the basis of M1. By calculating the PRS score of each individual under the M1 and M2 PRS models and performing a t-test analysis on the PRS score of the BC and NC groups, we found that the *P*-value (4.9*10^− 5^) of the M2 PRS model was far less than that of M1 (0.17). Moreover, the ROC curve (62.18%) of the M2 model was better than that of the M1 model (54.56%). Therefore, the model constructed by adding estrogen metabolic enzyme gene SNPs had a good ability in breast cancer risk prediction, and the accuracy was greatly improved.

There are several limitations of this study that should be noted. First, the sample size was relatively small. In this study, only 140 premenopausal women first diagnosed with BC and 140 matched healthy women were recruited based on our criteria; thus, we did not have enough statistical power to detect the effect of the genetic variants on some of the parameters. Second, because funding was limited, it did not include comprehensive metabolic enzymes and adequate breast cancer risk gene loci. Due to these reasons, the AUC was small and the model have not been tested. In the future, we will study additional estrogen-metabolizing enzyme genes and other breast cancer risk genes in our research. At the same time, we will also include recognized breast cancer risk factors such as age at evaluation, age at menarche, age at first live birth, race, number of breasts, and family history of breast cancer and construct a breast cancer risk prediction model composed of phenotype and genotype to obtain a more valuable ROC value. In addition, the sample size needs to be further expanded, and it is better to include more data information of different races.

## Conclusion

Estrogens and related metabolic enzyme gene polymorphisms are closely related to BC. The model constructed by adding estrogen metabolic enzyme gene SNPs has good predictive ability for breast cancer risk, and the accuracy is greatly improved compared with that of the PRS model that only includes GWAS-identified gene SNPs.

## Data Availability

The data that support the findings of this study are available from the corresponding author upon reasonable request.

## Abbreviations

BC: Breast cancer
BMI: Body mass index
CI: Confidence interval
COMT: Catechol-O-methyltransferase
CYP: Cytochrome P450
E1: Estrone
E2: 17β-estradiol
2-OHE2/1: 2-hydroxy estradiol/estrone
4-OHE2/1: 4-hydroxy estradiol/estrone
16α-OHE1: 16α-hydroxy estrone
2-MeOE2/1: 2-methoxy estradiol/estrone
4-MeOE2/1: 4-methoxy estradiol/estrone
ER: Estrogen receptor
ESI: Electrospray ionization source
FRR: Familial relative risk
GWAS: Genome-wide association study
HWE: Hardy–Weinberg equilibrium
LC-MS/MS: Liquid chromatography-tandem mass spectrometry
M1: PRS model 1
M2: PRS model 2
MRM: Multiple reaction monitoring
OPLS-DA: Orthogonal Partial Least Squares Discriminant Analysis
OR: Odds ratio
PRS: Polygenic risk score
QC: Quality control
ROC: Receiver operating characteristic curve
SNPs: Single nucleotide polymorphisms
SULTs: Sulfate transferases
UGTs: UDP-glucuronosyl-transferases
WHO: World Health Organization
